# Mutual influences of pain and emotional face processing

**DOI:** 10.3389/fpsyg.2014.01160

**Published:** 2014-10-13

**Authors:** Matthias J. Wieser, Antje B. M. Gerdes, Philipp Reicherts, Paul Pauli

**Affiliations:** ^1^Department of Psychology, University of WürzburgWürzburg, Germany; ^2^Department of Psychology, University of MannheimMannheim, Germany

**Keywords:** pain, emotion, facial expression, ERPs, perception-action

## Abstract

The perception of unpleasant stimuli enhances whereas the perception of pleasant stimuli decreases pain perception. In contrast, the effects of pain on the processing of emotional stimuli are much less known. Especially given the recent interest in facial expressions of pain as a special category of emotional stimuli, a main topic in this research line is the mutual influence of pain and facial expression processing. Therefore, in this mini-review we selectively summarize research on the effects of emotional stimuli on pain, but more extensively turn to the opposite direction namely how pain influences concurrent processing of affective stimuli such as facial expressions. Based on the motivational priming theory one may hypothesize that the perception of pain enhances the processing of unpleasant stimuli and decreases the processing of pleasant stimuli. This review reveals that the literature is only partly consistent with this assumption: pain reduces the processing of pleasant pictures and happy facial expressions, but does not – or only partly – affect processing of unpleasant pictures. However, it was demonstrated that pain selectively enhances the processing of facial expressions if these are pain-related (i.e., facial expressions of pain). Extending a mere affective modulation theory, the latter results suggest pain-specific effects which may be explained by the perception-action model of empathy. Together, these results underscore the important mutual influence of pain and emotional face processing.

## INTRODUCTION

Emotions possess immense power to alter pain perception. The influence of experimentally induced emotions on experimental pain has been investigated with various affective stimuli like affective pictures (e.g., Meagher et al., 2001; Rhudy and Meagher, 2001; Kenntner-Mabiala and Pauli, 2005; Kenntner-Mabiala et al., 2007, 2008), pain-related pictures (e.g., Godinho et al., 2012), odors (e.g., Villemure et al., 2003), and music (e.g., Roy et al., 2008, 2012). This research has demonstrated that negative emotions lead to increased pain perception while positive emotions result in decreased pain perception pain. Overall, it was found that emotions affect various measures of pain perception such as sensory and affective pain ratings, and neuronal correlates as measured with BOLD responses and EEG responses (for extensive reviews see Wiech and Tracey, 2009; Bushnell et al., 2013). Various brain mechanisms are implicated in the emotional modulation of pain (Roy et al., 2009; Wiech and Tracey, 2009). One key mechanism involves descending pain modulatory systems affecting the afferent transmission of spinal nociceptive signals to many brain regions (including the thalamus, amygdala, insula, and somatosensory cortex), which can lead to inhibition or excitation of afferent pain signals (Bushnell et al., 2013). Other mechanisms involve the integration of pain- and emotion-related signals in the anterior insula, also with regards to signals from the autonomic nervous system which are central to both pain and emotions (Craig, 2002). Overall, the multiplicity of mechanisms underlying the emotional modulation of pain is reflective of the strong interrelations between pain and emotion both on a psychological and neuroanatomical level (Vogt, 2005; Roy et al., 2009).

A theoretical framework for the explanation of the emotional modulation of pain is the motivational priming hypothesis (Lang, 1995), which assumes that processing of unpleasant information is facilitated while processing of pleasant information is inhibited under aversive affect. Accordingly, unpleasant stimuli increase and pleasant stimuli decrease pain perception and physiological responses to pain. Interestingly, the majority of studies on emotion-pain interactions has unidirectionally examined the influence of emotional stimuli on pain perception, although bidirectional interactions are likely and plausible (Bushnell et al., 2013). This would also concur with predictions drawn from the motivational priming hypothesis that pain as an aversive state should facilitate the processing of unpleasant stimuli and inhibit the processing of pleasant stimuli.

As mentioned above and also common in emotion research, most of the studies investigating emotional modulation of pain used affective pictures (e.g., Meagher et al., 2001; Rhudy and Meagher, 2001; Kenntner-Mabiala and Pauli, 2005; Kenntner-Mabiala et al., 2007, 2008) or pain-related pictures (e.g., Godinho et al., 2012). Only recently, researchers started to investigate the effects of facially communicated pain stimuli (i.e., facial expressions of pain), their expression, perception, and possible effect on pain perception. While in general it is doubtful that facial expressions elicit strong emotional states in the observer (e.g., Bradley et al., 2001; Britton et al., 2006; Alpers et al., 2011) the social importance of non-verbal emotion communication makes facial expressions (of pain) an interesting model for the interaction of facial signals of emotions and concurrent pain processing (Williams, 2002). However, the modulation of pain by this crucial feature in non-verbal emotion communication has been widely neglected so far. One of the few studies in this field demonstrated that emotional compared to neutral facial expressions increase pain perception accompanied by alterations of pain-related brain oscillations (Senkowski et al., 2011). Similarly, pain compared to neutral expressions were found to augment pain perception (Mailhot et al., 2012). However, the effect of pain on pain face processing was not quantified.

A possible theoretical explanation for the interaction of viewing others’ facial expression of pain and the own sensation of pain is offered by the perception-action model (PAM) of empathy (Preston and de Waal, 2002). The PAM proposes that the capacity to feel the internal state of someone else activates the corresponding representations in an observer. Indeed, it was found that observing others’ facial expression of pain also amplifies one’s own facial and neural responses to pain, revealing a vicarious effect of facial pain expression (Vachon-Presseau et al., 2011, 2013; Mailhot et al., 2012). Additional support for the PAM derives from neuroimaging studies in which it was found that emotions observed in a target are mapped onto a self-reference framework supposed to serve the rapid understanding of others’ feelings, goals, and intentions (e.g., Wicker et al., 2003; Jackson et al., 2006). Consequently, the PAM would predict selective pain-enhancement by watching pain faces of others compared to other negative facial expressions, whereas the motivational priming would assume a general enhancement of pain by negative facial expressions, but not necessarily selectivity of pain faces.

The aim of this mini-review is to selectively summarize research on the influence of visual affective stimuli on pain perception, but mainly on the opposite effect of pain on the processing of affective stimuli such as facial expressions. Given the growing interest in pain modulation by facial expressions of pain and the lack of studies which used other affective stimuli, we focus on studies on facial expressions. In addition we seek to extend the viewpoint of a mere affective modulation of pain with regards to the theory of vicarious pain and include a recent experiment from our lab which aimed at investigating the mutual effects of the perception of facial expressions of pain and pain perception. This review is far from exhaustive; it only summarizes the literature relevant for our work within the research group “Emotion and Behavior” at the University of Würzburg, Germany. We are fully aware that much more research is available on the topics of emotional modulation of pain and vicarious pain, and we direct the attention of the interested reader to the excellent reviews by Wiech and Tracey (2009), Lamm et al. (2011) and Bushnell et al. (2013).

## PAIN-MODULATED PROCESSING OF AFFECTIVE PICTURES AND FACIAL EXPRESSIONS

As mentioned above, the effect of pain on emotion processing has been investigated much less, although from a clinical perspective the high prevalence of mood disorders in chronic pain suggests effects in this direction (Bair et al., 2003; Campbell et al., 2003). One study found that when paired with pain, pleasant pictures were rated less pleasant and elicited attenuated visual-evoked responses of the EEG (Godinho et al., 2008). However, no enhanced responses to negative stimuli were found. In an own study, evaluative facial responses congruent and incongruent to pictures of facial expressions were recorded during painful pressure stimulation (Gerdes et al., 2012). Normally, voluntary facial muscle reactions are facilitated (i.e., less errors and faster responses) in response to muscle-congruent facial expressions (i.e., facilitated reactions of Musculus Corrugator supercilii in response to negative facial expressions and facilitated reactions of Musculus zygomaticus major in response to positive facial expressions), which is interpreted as motor-compatibility and automatic evaluation of affective stimuli. In this study, pressure pain generally slowed compatible as well as incompatible muscle responses (Musculus zygomaticus and Musculus corrugator) and resulted in fewer erroneous incompatible (Musculus corrugator) responses to happy faces. However, pain did not affect muscle responses to angry faces and affective ratings. Together with the results by Godinho these findings point at the notion that pain particularly reduces responses to pleasant stimuli, but seems not necessarily to exacerbate processing of negative emotional stimuli. The latter observation may be partly explained by the pain-reducing effects of distraction which is caused by the ongoing pain and thus dampens the actual facilitatory effects of pain for unpleasant emotions.

In a further study, we then investigated the effect of tonic pressure pain on the electrocortical correlates of processing of facial expressions (Wieser et al., 2012). Here, fearful, happy, and neutral faces were presented while participants received tonic pressure stimulation. Face-evoked brain potentials revealed no affective but an attentional modulation by pain: early and late indices of attention allocation toward faces [P100 and late positive potentials (LPP) of the ERP] were diminished during tonic pain compared to the control condition. The latter finding is concurrent with earlier findings revealing the attentional interruptive function of pain (Eccleston and Crombez, 1999), which has been demonstrated for attentional processes (e.g., Seminowicz and Davis, 2006; Tiemann et al., 2010), visual object processing (Bingel et al., 2007), and early stages of memory formation (Forkmann et al., 2013).

We conclude that on the one hand there is some evidence that experimental pain alters perception and processing of positive affective stimuli (scenes and faces), although most effects were observed with regards to attentional mechanisms. On the other hand, little is known about how pain alters processing of facial displays of pain and vice versa. Given the match between observed and experienced pain, one may argue that selective enhancement and mutual influences have to be expected. Before we report an experiment in which these mutual influences were investigated, we will briefly summarize why facial expressions of pain may be special compared to other facial expressions.

## PAIN-SPECIFIC MODULATIONS: FACIAL EXPRESSIONS OF PAIN, THEIR PERCEPTION, AND THEIR EFFECT ON PAIN

The sensation of pain is accompanied by distinct albeit not uniform facial expressions (e.g., Prkachin, 1992; Prkachin and Craig, 1995; Kunz et al., 2009, 2011; Kunz and Lautenbacher, 2014). Pain expressions, on the one hand, may benefit the sender by observers’ support and assistance in recovery, and on the other hand, inform observers about potential threat and danger (Williams, 2002). Thus, facial displays of pain serve important social functions and therefore are supposed to be of great importance for social interactions (Williams, 2002; Craig, 2009).

**FIGURE 1:**
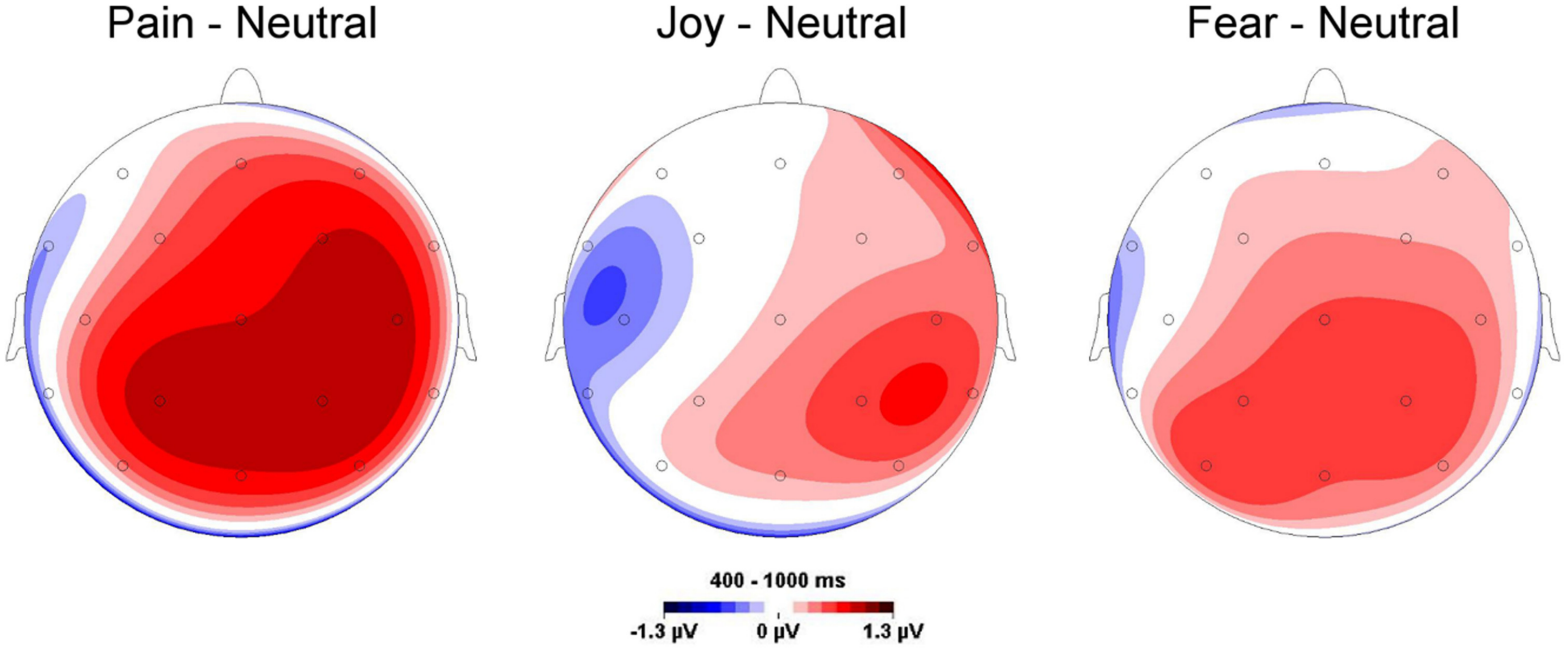
**Topographies for mean late positive potential (LPP) amplitude differences elicited by emotional and neutral facial expressions.** Pronounced LPPs are particularly found for painful compared to neutral facial expressions. Data are taken from Reicherts et al. (2012).

Compared to neutral facial expressions, facial expressions of pain receive prioritized processing and elicit enhanced initial orienting (Vervoort et al., 2013). Similar results were obtained in a dot-probe paradigm (Baum et al., 2013), whose results indicated both early attentional engagement and subsequent avoidance of facial expressions of pain. A recent study from our lab investigated whether facial expressions of pain are perceived differentially from other facial expressions and elicit distinct electro-cortical responses as measured by ERPs (Reicherts et al., 2012). To this end, participants watched painful, fearful, happy, neutral facial expressions (Simon et al., 2008) and were asked to rate these videos while EEG was recorded continuously. Videos of pain faces were rated as more intense and negative than other emotional (both positive and negative) expressions and concurrently elicited enhanced electrocortical responses (augmented late positive potentials, LPPs), which are supposed to index sustained motivated attention to salient stimuli (Schupp et al., 2000) (see **Figure 1**).

Corroborating our findings, Missana et al. (2014) reported enhanced LPPs of pain compared to angry faces. Additionally, two studies observed that pain faces were rated as more unpleasant and arousing than angry, happy, and neutral faces, resulted in enhanced amplitudes of the ERPs and increased theta activity, and evoked greater corrugator response (González-Roldán et al., 2011, 2013). Taken together, these results emphasize that pain faces have a special salience different from other emotional expressions, although one has to bear in mind that in none of the aforementioned studies a comparison was made between all facial expressions (e.g., facial expressions of disgust) within one study.

Since emotional stimuli have a great impact on pain perception as summarized above, one may assume that a highly salient signal such as the facial expression of pain should also modulate pain perception. This assumption is further strengthened by the fact that the cortical regions involved in the decoding of pain (Bushnell et al., 1999) and emotional facial expressions (Adolphs et al., 2000), e.g., the somatosensory cortex, partly overlap.

The interaction of facial expressions of pain and perception of pain is rarely investigated. In one study, volunteers viewed videos showing different levels of pain expression before noxious electric shocks were delivered. Viewing stronger pain expressions generally increased pain unpleasantness ratings, the amplitude of the nociceptive flexion reflex, and corrugator responses to the noxious stimulation (Mailhot et al., 2012). In another study, the influence of pictures displaying noxious stimulation to the foot or hand of others compared to pain faces on pain perception was investigated (Vachon-Presseau et al., 2011). Again, enhanced pain perception was found when participants viewed others’ pain, however, more robust facilitation of pain perception was found in response to images of noxious stimulation to the foot and hand compared to facial expression of pain (Vachon-Presseau et al., 2011). Roy et al. (2013) recently demonstrated that characteristic visual features of pain expressions are sufficient to induce this enhanced pain perception. Together, these studies confirm the augmentation of pain responses when observing pain in others, although no control for other (negative) facial expressions was used and hence it remains unclear whether this effect is specific to facial expressions of pain.

Besides the lack of information of the pain-specificity of these effects, little is known to date about the mutual effects of the perception of facial expressions of pain in others and the own pain sensations. Given the observations from functional neuroimaging that seeing others facial expression of pain leads to activations in pain-related areas in the brain of the observer, one may assume strong interactions between seeing pain of others and feeling pain. A number of studies using methods such as functional magnetic resonance imaging and electrophysiological recordings have provided support for this view by showing increased activity in pain-related brain regions during perception of pain in others (Fan et al., 2011; Lamm et al., 2011) including facial expressions of pain (Vachon-Presseau et al., 2012).

**FIGURE 2:**
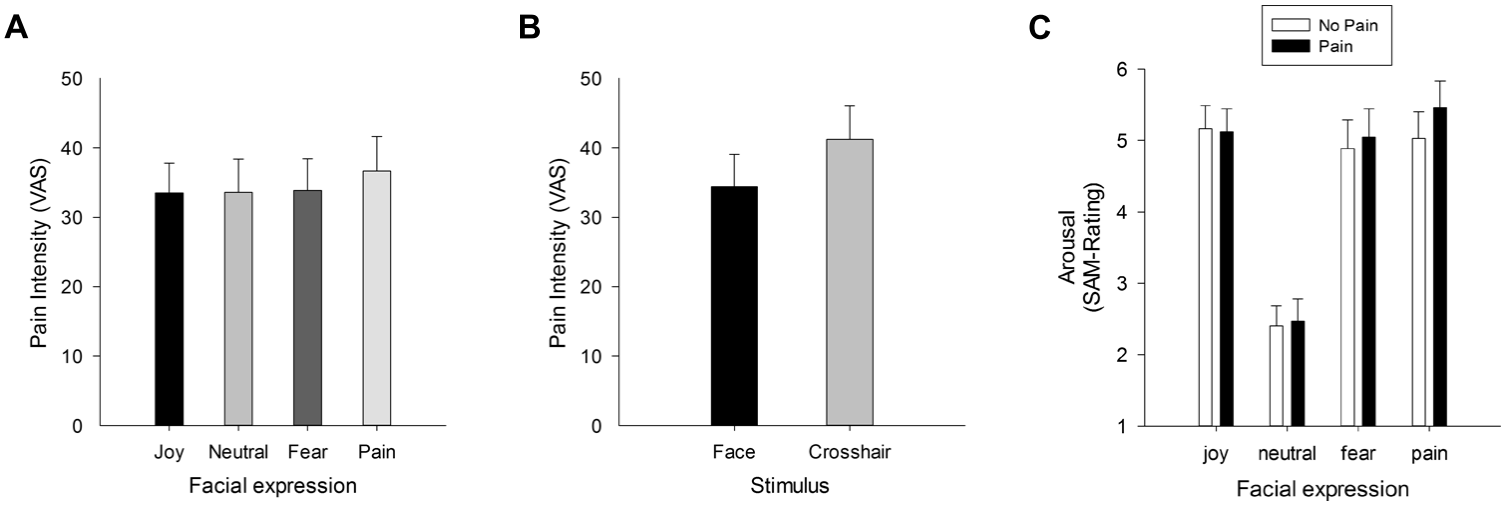
**Pain intensity ratings (VAS 0 = no pain – 100 = extreme pain) when watching facial expressions **(A)**, and collapsed across facial expressions compared to trials with crosshairs **(B)**.** Pain-specific enhancement of pain perception is shown in **(A)**, whereas **(B)** depicts the attentional effect of watching complex social stimuli such as faces compared to low-level visual stimulation (crosshairs) which results in reduced pain. Data are taken and figure is adapted with permission from Reicherts et al. (2013). In **(C)**, the arousal ratings for facial expressions are given, when participants experienced painful and non-painful thermal heat. The figure has been reproduced with permission of the International Association for the Study of Pain^®;^ (IASP). The figure may not be reproduced for any other purpose without permission.

In a recent study we aimed at investigating both the effects of facial expressions of pain on the actual perception of pain, and vice versa the influence of pain sensation on the affective ratings of facial expressions of pain (Reicherts et al., 2013). To this end, participants received painful thermal stimuli while passively watching dynamic facial expressions (joy, fear, pain, and a neutral expression). To compare the influence of complex visual with low-level stimulation, a central fixation cross was presented as control condition. Participants were asked to rate the intensity of the thermal stimuli and also to evaluate valence and arousal of the facial expressions. In addition, facial electromyography was recorded as an index of emotion and pain perception. Results show that faces in general compared to the low-level control condition decreased pain ratings, suggesting a general attention modulation of pain by complex (social) stimuli. In addition, the facial response to painful stimulation was found to correlate with pain intensity ratings. Most important, painful thermal stimuli increased the perceived arousal of simultaneously presented fear and especially pain expressions of others, and vice versa, pain expressions of others compared to all other facial expressions led to higher pain ratings. The independent effects of attention and facial expressions on pain ratings are depicted in **Figures 2A,B**, the selective enhancement of arousal ratings of pain faces by pain in **Figure 2C**.

These findings demonstrate that the relation between pain and emotion is bidirectional with pain faces showing selectively mutual influences. This study provides further experimental evidence that processing painful stimulation and the facial expressions of pain in others are highly interconnected. Extending previous findings it also shows pain-specific modulations of pain perception such that highest pain ratings of painful thermal stimuli were obtained while participants watched faces of pain compared to other facial expressions. Importantly, this effect was also larger for pain compared to fear faces, suggesting that the facial expressions of pain enhance self-pain perception not only due to its negative valence but due to its pain relevance. In addition to the predictions from the motivational priming theory, these results support the notion that not only the valence of a facial expressions enhances pain perception, but that the expressed pain itself primes the sensorimotor system, which might drive a potentiating pro-algesic mechanism (Godinho et al., 2012). Additional evidence for this amplification of pain driven by the perception of others’ pain comes from several studies showing that watching facial pain expressions results in augmented pain ratings (Vachon-Presseau et al., 2011, 2012, 2013; Mailhot et al., 2012).

Another potential mechanism of pain modification in addition to the affective priming hypotheses has been put forward as the PAM of empathy (Preston and de Waal, 2002). This model would postulate that the observation of others’ pain activates a similar neural network implicated in the first-person experience of the very phenomenon (Jackson et al., 2005). Accordingly, the perceived pain expression of others is mapped on the observer’s own neural representations and as such facilitates and primes own-pain perceptions. This shared representations account has been supported by neuroimaging studies (e.g., Jackson et al., 2006). However, it has to be noted that the brain responses to pain and to facial expressions of pain may not indicate shared representations of actual pain and observed pain, but a much more unspecific response to salient stimuli (Iannetti et al., 2013).

This mini-review featured recent work on the emotional modulation of pain perception by affective stimuli such as facial expressions, but more importantly on the reverse impact of pain on emotional face processing. The presented studies also further underscore the special relevance of facial expressions of pain. The functional significance of pain faces for human social interaction, however, is still under debate, therefore future work needs to clarify whether they elicit predominantly approach or avoidance behavior in the observer (Yamada and Decety, 2009; Hadjistavropoulos et al., 2011). This would probably be accomplished best by incorporating measures of behavioral consequences (Krieglmeyer et al., 2013). The most recent study in our research program aimed at combining methods investigating mutual influences of pain and emotion processing. These results revealed exciting insights on how pain and facial expressions of pain may interact. Clearly, future research along the aforementioned theories should clarify the specificity of pain enhancement due to pain faces but also further elucidate the common neural substrates of pain and facial emotion processing.

## Conflict of Interest Statement

The authors declare that the research was conducted in the absence of any commercial or financial relationships that could be construed as a potential conflict of interest.
